# Comparative analysis of hepatitis C virus phylogenies from coding and non-coding regions: the 5' untranslated region (UTR) fails to classify subtypes

**DOI:** 10.1186/1743-422X-3-103

**Published:** 2006-12-14

**Authors:** Peter T Hraber, William Fischer, William J Bruno, Thomas Leitner, Carla Kuiken

**Affiliations:** 1 Theoretical Biology and Biophysics, T-10 MS K710, Los Alamos National Laboratory, Los Alamos NM 87545 USA

## Abstract

**Background:**

The duration of treatment for HCV infection is partly indicated by the genotype of the virus. For studies of disease transmission, vaccine design, and surveillance for novel variants, subtype-level classification is also needed. This study used the Shimodaira-Hasegawa test and related statistical techniques to compare phylogenetic trees obtained from coding and non-coding regions of a whole-genome alignment for the reliability of subtyping in different regions.

**Results:**

Different regions of the HCV genome yield inconsistent phylogenies, which can lead to erroneous conclusions about classification of a given infection. In particular, the highly conserved 5' untranslated region (UTR) yields phylogenetic trees with topologies that differ from the HCV polyprotein and complete genome phylogenies. Phylogenetic trees from the NS5B gene reliably cluster related subtypes, and yield topologies consistent with those of the whole genome and polyprotein.

**Conclusion:**

These results extend those from previous studies and indicate that, unlike the NS5B gene, the 5' UTR contains insufficient variation to resolve HCV classifications to the level of viral subtype, and fails to distinguish genotypes reliably. Use of the 5' UTR for clinical tests to characterize HCV infection should be replaced by a subtype-informative test.

## Background

In treating infection with hepatitis C virus, knowledge of a patient's viral genotype informs the choice of appropriate therapy [1-3]. Although the HCV subtype afflicting a patient is not currently used to make clinical treatment decisions, knowing the viral subtype is important for studies of its origin, transmission, and evolution [1-4]. For example, new emerging variants can be characterized better when they can be assigned an unequivocal subtype classification [5]. Molecular epidemiology analyses rely on information about sequence variation at the subtype level [4,5]. Vaccine-design strategies are informed by the diversity of HCV variants and the antigenic determinants (epitopes) therein [6,7]. The risk of hepatocellular carcinoma, a frequent complication for HCV infection, might be assessed better in light of HCV subtype [8]. Thus, effective methods for both genotype and subtype classification are important tools to manage HCV infections.

Techniques to infer phylogenies combine an optimality criterion with an algorithm to search for the best tree. Optimality criteria quantify how well the tree describes the data, and are either distance-based or character-based [9,10]. An algorithm can quickly construct a single tree that minimizes all the pairwise distances among taxa. However, this approach is less able to use information from different taxa to model variation in evolutionary rates across sites than the optimality criterion of maximum likelihood ([9], p. 175). Search algorithms are deployed by character-based methods to find trees that best explain the data, given an evolutionary model with known assumptions. The search algorithms of character-based methods take more time to evaluate alternative candidate trees than rapid distance-based methods. Perhaps for this reason, many more distance-based than character-based phylogenies of HCV genotypes have been published. However, maximum-likelihood phylogenetic inference is known to outperform distance-based methods when such complications as substitution rate heterogeneity or covariation between sites are present [9,10]. Formal comparisons between topologies are thus more appropriate for maximum-likelihood phylogenies than for the approximations that result from distance-based methods.

This study evaluates phylogenies derived from coding (NS5B) and non-coding (5' UTR) regions of whole-genome HCV sequences for consistent classification of viral subtypes into distinct genetic groups, or clades, with the aim of evaluating their suitability for genotype and subtype classification. Concordance with the whole-genome phylogeny is desired. Nucleotide characters in NS5B are over five times more abundant than in the 5' UTR, though only a small portion of this region is amplified for subtyping. To compensate for this, we also considered a smaller, oft-studied portion of NS5B that we call the "Okamoto region" (from nt 8282 to 8610 in the H77 reference genome) for its ability to represent the phylogeny of NS5B and the entire HCV genome. We tested the hypothesis that phylogenetic trees obtained from different genomic regions of HCV differ significantly. We also compared tree topologies for their ability to group genotypes and subtypes consistently into clades.

## Results

### Phylogenetic inferences

Among the 38 whole-genome HCV sequences representing 18 confirmed subtypes as summarized in Table 1, the most general substitution model, the general time reversible model (GTR, also known as REV) with a discrete gamma approximation for rate heterogeneity, was consistently supported as superior among the twelve nucleotide substitution models evaluated (not shown). Models adjusted for rate heterogeneity consistently fit the data better than models that assume a fixed evolutionary rate across sites (not shown). Substitution models with fewer parameters or an assumption of equal base compositions performed significantly worse than GTR, regardless of whether or not the sequences analyzed contained protein-coding regions. Adding a parameter for the estimated proportion of invariant sites significantly improved the substitution model, yielding parameters as shown in Table 2. The same model was selected when the AIC was adjusted to compensate for a low ratio of sample data to parameters (not shown). Thus, GTR with a gamma distribution of evolutionary rates per site and accommodation of invariant sites (GTR+Γ+I) is the best substitution model for HCV variation among those considered, and was used for maximum-likelihood phylogeny inference.

**Table 1:** Confirmed subtypes and accession numbers of HCV genomes studied.

**Subtype**	**Database Accession Numbers**
1a	[EMBL:AF009606, EMBL:AF511950, EMBL:D10749, EMBL:M62321]
1b	[EMBL:AF483269, EMBL:AJ000009, EMBL:D11168, EMBL:L02836]
1c	[EMBL:AY051292, EMBL:AY651061, EMBL:D14853, EMBL:E08443]
2a	[EMBL:AB047639, EMBL:AF169003, EMBL:AF169005, EMBL:D00944]
2b	[EMBL:AB030907, EMBL:AF238486, EMBL:AY232746, EMBL:D10988]
2c	[EMBL:D50409]
2k	[EMBL:AB031663]
3a	[EMBL:AF046866, EMBL:D17763, EMBL:D28917, EMBL:X76918]
3b	[EMBL:D49374, EMBL:E10840]
3k	[EMBL:D63821]
4a	[EMBL:Y11604]
5a	[EMBL:Y13184]
6a	[EMBL:AY859526, EMBL:Y12083]
6b	[EMBL:D84262]
6d	[EMBL:D84263]
6g	[EMBL:D63822]
6h	[EMBL:D84265]
6k	[EMBL:D84264]

**Table 2:** Substitution model (GTR+Γ+I) parameters and alignment properties.

**Model Parameter**	**Genome**	**Polyprotein**	**5' UTR**	**Okamoto**
A proportion	0.2034	0.2046	0.1920	0.288
C proportion	0.3261	0.3267	0.2913	0.3302
G proportion	0.2675	0.2698	0.3081	0.2667
U proportion	0.2030	0.1989	0.2086	0.1743
A-C rate	1.6280	1.5920	16.9081	1.2156
A-G rate	5.9755	5.8823	56.7130	3.5749
A-U rate	2.7662	2.7764	54.5047	1.3329
C-G rate	1.1295	1.1087	4.7757	0.5330
C-U rate	7.5166	7.5910	128.7054	5.4729
G-U rate	1.0000	1.0000	1.0000	1.0000
Proportion of invariant sites (I)	0.2693	0.2549	0.6637	0.2881
Γ-distribution shape parameter	0.8357	0.8601	0.9055	1.3298
Nucleotides in alignment	9791	9177	300	329
Conserved sites in alignment	3473	3028	251	223

The 5' UTR is represented by the smallest number of aligned nucleotide sites (300 nt; the 5' most 42 nt were excluded from analysis because of extensive gaps throughout the available sequence data), followed by the Okamoto region of NS5B (329 nt), then the polyprotein (9177 nt), and the whole genome (9791 nt, Table 2). The proportion of invariant nucleotide sites for the 5' UTR is 2/3, much lower than for the protein-coding regions, for which less than 1/3 of sites do not vary (Table 2). The 5' UTR is known to be less variable than protein-coding regions of HCV [3,6,11,12].

**Figure 1:**
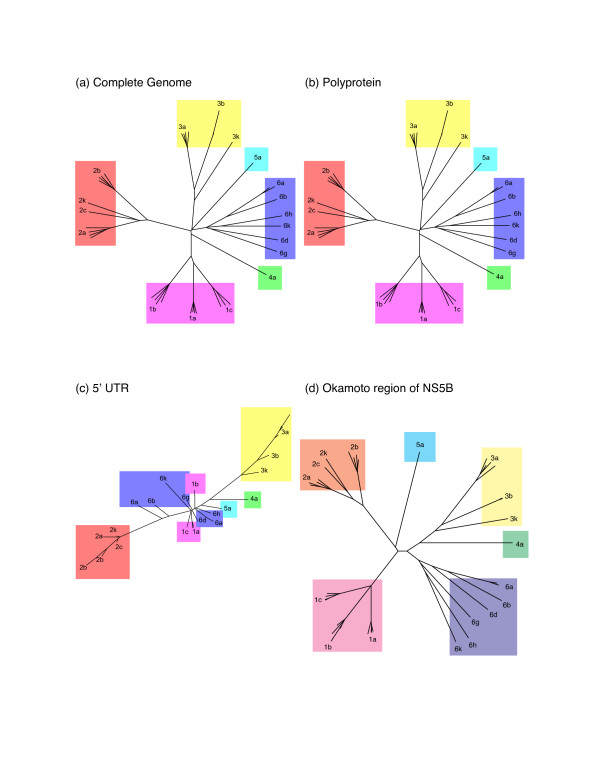
**Neighbor-joining phylogenies**. Unrooted neighbor-joining phylogenetic trees from (a) complete HCV genome, (b) polyprotein, (c) 5' UTR, and (d) the Okamoto region of NS5B. Due to our focus on the consistency of subtype classification and the relative branching topology among subtypes, each tree is scaled independently.

**Figure 2:**
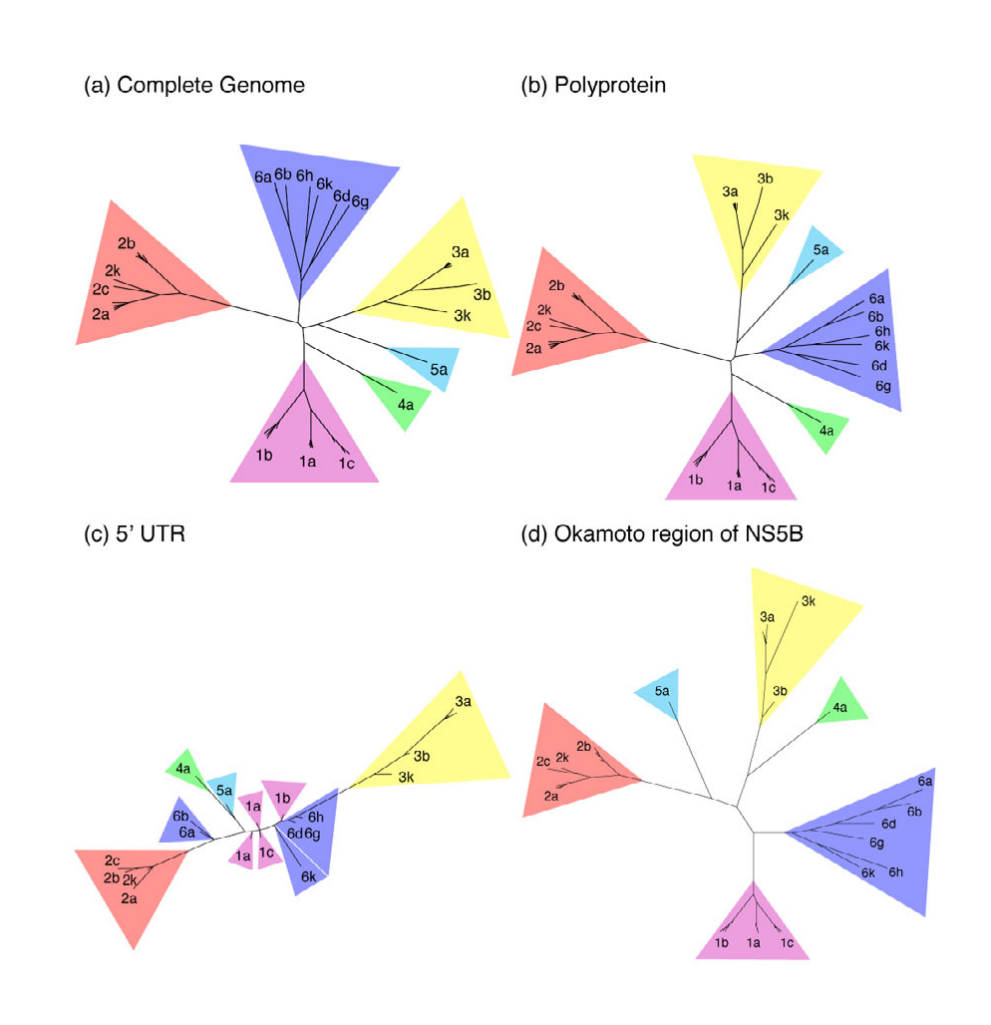
**Maximum-likelihood phylogenies**. Unrooted maximum likelihood phylogenetic trees from (a) complete HCV genome, (b) polyprotein, (c) 5' UTR, and (d) the Okamoto region of NS5B. Taxon labels indicate HCV genotype and subtype from Table 1. Due to our focus on the consistency of subtype classification and the relative branching topology among subtypes, each tree is scaled independently.

Tree topologies from the entire HCV genome and the polyprotein are identical (Figs. 1a, b and 2a, b). The tree from the Okamoto region of NS5B resembles trees from the whole genome and the polyprotein, except for rearrangements in the ordering of deeply rooted branches (Figs. 1d and 2d). Trees from sequences that include protein-coding regions clearly group subtypes from the same genotype into clades, while the tree from the non-coding terminus conflates subtypes of genotypes 1 and 6 with subtypes 4a and 5a, and subtypes of genotypes 1 and 6 cannot be distinguished (Figs. 1c and 2c). Thus, the phylogenetic trees of the 5' UTR are less able to group subtypes from the same genotype together into clades than trees from protein-coding sequences (Figs. 1 and 2), regardless of the method used for phylogenetic inference. Parsimony analysis yields comparable results, with similar trees for the whole genome, polyprotein, and the Okamoto region of NS5B, while the tree from the 5' UTR contains a basal polytomy that does not resolve genotypes 1,4, 5, or 6 (not shown).

### Hypothesis tests

Log-likelihood scores and SH-test results for alternative trees are summarized in Table 3. All tests yield the same outcomes, regardless of whether or not RELL optimization was used. Comparisons of alternative trees with the 5' UTR data fail to reject the null hypothesis of no difference in likelihoods (P > α; see Methods). Comparisons among alternative trees with data from the Okamoto region of NS5B indicate that the 5' UTR tree has a significantly different likelihood (P < 0.0001) than trees obtained from NS5B, polyprotein, or whole-genome data, which are statistically indistinguishable (P > α). Comparing parsimony trees from 300-nt windows in NS5B with trees from the 5' UTR via the incongruence length difference test [13], which uses the difference in tree lengths as a test statistic, rather than the likelihood difference, yielded the same pattern of significant differences (not shown).

**Table 3:** Shimodaira-Hasegawa test results from 10,000 bootstrap replicates.

**Tree**	**-ln *L***	**-ln Δ**	**P_RELL_**	**P_FULL_**
**5' UTR sites**				

5' UTR	895	0	--	--
Whole genome	955	61	0.0225	0.0153
Polyprotein	956	62	0.0221	0.0144
Okamoto region	949	54	0.0323	0.0215

**Okamoto region sites**				

Okamoto region	5,226	0	--	--
Whole genome	5,256	30	0.2824	0.2872
Polyprotein	5,255	29	0.2981	0.3050
5' UTR	5,898	672	< 0.0001	< 0.0001

### Consistency and homoplasy indices

**Figure 3:**
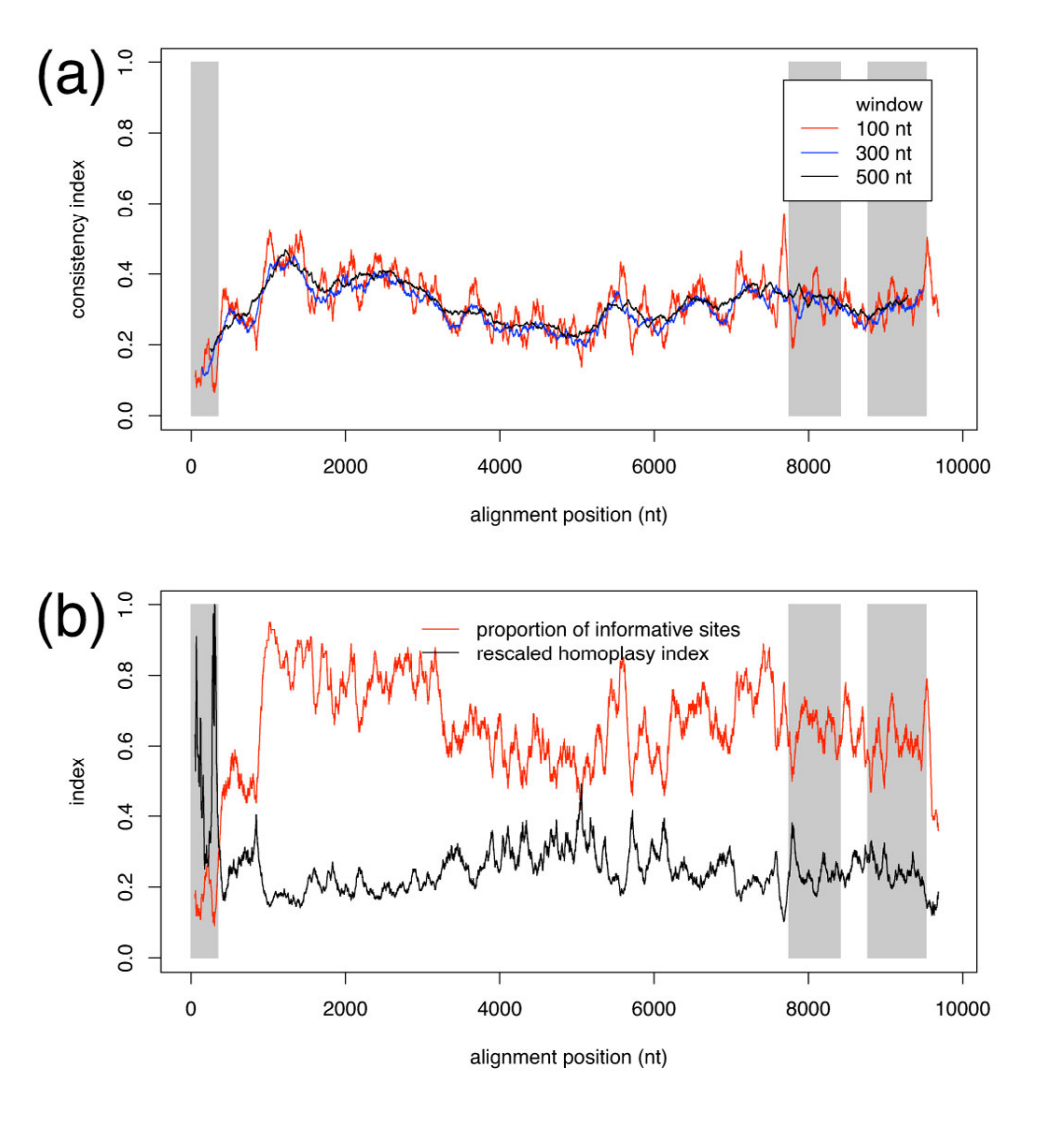
**Consistency and homoplasy indices**. Moving-window averages of (a) character consistency with the whole-genome phylogeny for windows of 100 (red), 300 (blue), or 500 (black) nucleotides and (b) proportion of informative sites (red) and rescaled homoplasy index (black) for windows of 100 nucleotides as a function of the window midpoint in the whole-genome alignment. Regions corresponding to the 5' UTR (left) and NS5B (right) are indicated with grey bands, with a white band in the middle of NS5B to indicate the 329 nt Okamoto region.

Increasing window sizes represent the CI as an increasingly smooth function, as more nucleotides better approximate the whole-genome phylogeny than fewer nucleotides. However, increasing window size yields poorer resolution in the 5' UTR (Fig. 3a) because fewer windows are able to represent this region. Contrary to expectations, the rescaled homoplasy index is not constant. Despite large fluctuations within the 5' UTR, the rescaled homoplasy index is generally greater in the 5' UTR than in other regions of the HCV genome and particularly NS5B (Fig. 3b). After correcting for the substitution rate in this manner, the consistency of sites with the whole-genome phylogeny is lower in the 5' UTR than in NS5B.

## Discussion

An earlier investigation of phylogenetic relations among 27 complete HCV genomes used maximum likelihood and careful determination of the appropriate nucleotide substitution model, and reported a star-like phylogeny among the six known HCV genotypes [12]. The best substitution model was also found to be the most general. In the earlier study, the 5' UTR was found to have lower phylogenetic signal, lower evolutionary rate, and greater phylogenetic noise than alternative regions of the HCV genome, including NS5B [12]. Our observations concur with those previously reported. Methodological refinements in our approach include the use of information-based model selection criteria to determine the best nucleotide substitution model, more complete HCV genomes, the revised nomenclature for subtypes [5], and formal comparisons between alternative topologies for the purpose of subtype determination.

The tree from the Okamoto region of NS5B is a significantly better fit to the HCV whole-genome and polyprotein data than the 5' UTR tree, regardless of the optimality criterion used for phylogenetic inference. Trees obtained from the 5' UTR perform worse at classifying HCV subtypes into clades of the same genotype than do trees from the whole genome, polyprotein, or the Okamoto region of NS5B. Discordant topologies of maximum-likelihood phylogenetic trees obtained from the 5' UTR and NS5B have been described for a subset of HCV genotypes [14,15]. The inconsistent ordering of deeply rooted branches among trees from protein-coding regions indicates a basal polytomy whose resolution is contingent on the data available, which accords with the star-like phylogeny of all six known HCV genotypes previously reported elsewhere [3,5,12,16].

The same evolutionary model (GTR with a discrete-gamma distribution of rate variation) used here has been utilized previously for likelihood phylogenies of the hepatitis B virus [17] and, with accommodation of invariant sites, for both HIV [18] and HCV [12]. Instantaneous substitution rates (normalized to the G-U rate) are greater among sites in the non-coding 5' UTR than in the regions that encode proteins, despite the fact that overall sequence conservation is greater in the UTR (Table 2). In particular, the instantaneous substitution rate between cytidine and uridine is much greater for the 5' UTR than for protein-coding regions. The accelerated C-U (or C-T for DNA sequences) substitution rate has previously been reported and discussed for protein-coding regions [19], though the rate is even greater for the non-coding terminus than for regions having codon usage constraints. Spontaneous deamination of cytosine to uracil may inflate the C-U substitution rate.

Conservation of single-stranded RNA secondary structure in both coding and non-coding regions of HCV has already been reported [15,20-23]. The high C-U rate bias may additionally be explained by the formation of non-canonical base pairs between guanosine and uridine in single-stranded RNA molecules, which is consistent with selection to conserve secondary structure, because a mutation from cytosine to uridine is less disruptive to secondary structure formation than other point mutations [24]. The may also be explained by the fact that all rates are rescaled such that the G-U rate is unity. A low G-U substitution rate thus inflates other rates. A mutation between G and U is disruptive to RNA secondary structure, because it eliminates the possibility of bases pairing without a compensatory mutation elsewhere. Overall, the elevated C-U substitution rate seen for the 5' UTR probably results from several interacting factors.

Though the same evolutionary model applies to the non-coding 5' UTR and the Okamoto region of NS5B, the two regions are subjected to different constraints. While coding sequences have codon-usage constraints and selective pressure for amino-acid mutations to escape detection by the host immune system, the UTR must preserve long-range interactions with complementary nucleotides at the other terminus of the viral genome if cyclization of the genome is essential to viral replication [6,20]. Because of these differences in selective regimes, it should not be surprising that phylogenies of the two differ.

HCV diagnostic technologies include serologic (antibody based) and genetic (sequence based) techniques to detect infected samples [4,6,25]. Population screens are the most commonly deployed genetic HCV tests, which benefit from low false-positive rates because they utilize the conserved 5' UTR as targets for PCR amplification. However, it is clear both from the results of this study and from previous investigations that the 5' UTR does not contain sufficient information to resolve subtypes [26-31]. Phylogenetic signal in protein-coding regions, such as NS5B, provides a useful alternative [12,32], but few commercial assays exploit this information at present. The "gold standard" for subtype determination is direct sequencing, which has a lower cost for reagents but requires more time than commercial assay kits [4,25].

There exist further complications to subtype classification, including coinfection [30,33,34], recombination [35,36], within-host evolution [37,38], and compartmentalization of genotypes into different cell types [39]. Diagnostic assays that are informed by the 5' UTR will be less able to accommodate these difficulties than methods that are able to resolve subtypes.

## Conclusion

Ultimately, HCV infection outcome results from an interaction between the virus and its host. The current standard of care is limited in efficacy, and treatment outcome is contingent on viral genotype [1-3,6,25,34]. To improve HCV therapies, perform effective public-health surveillance for new variants and modes of transmission, and further vaccine development efforts, detailed information about the interacting genotypes is needed. Diagnostic methods that assign viral subtype classifications are thus greatly desired. Such methods perform better when they are not informed by sequence variation from the non-coding 5' UTR, and should instead favor protein-coding regions, such as the Okamoto region of NS5B.

## Methods

### Phylogenetic inference

We used multiple methods for phylogenetic inference, including neighbor joining (NJ), maximum parsimony (MP), and maximum likelihood (ML) [9,10]. This was done to evaluate whether the inferential technique has an influence on the ability of the resulting phylogenies to resolve subtypes into clades. We used PAUP*, version 4.0b10 [40] for phylogenetic inference. Neighbor-joining trees were constructed with the F84 distance metric [41] and the BioNJ algorithm [42]. For parsimony analyses, uninformative invariant characters were excluded and gaps were treated as a fifth character state.

To select an appropriate nucleotide substitution model, we used FindModel, an independ-ent, online implementation of ModelTest [43]. This approach uses an information-based goodness-of-fit criterion, in the sense that the best model minimizes the quantity of bits required to encode both the model and the model-encoded data for electronic transmission [44-46]. Such an approach includes a penalty term for the number of parameters, and thus facilitates comparing models with varied numbers of parameters [44]. The fit of each model to the data was evaluated both with and without a four-category discrete approximation to a gamma distribution of substitution rates per site. Because FindModel does not test models with invariant sites, we also used ModelTest (version 3.6) to evaluate nucleotide substitution models with invariant sites [43]. Akaike's information criterion (AIC) was used to quantify the suitability of alternative models having varied numbers of parameters to fit the data [47].

### Hypothesis tests

To evaluate the significance of differences in ML phylogenies obtained from different regions of the HCV genome, we used the Shimodaira-Hasegawa (SH) test [48] as implemented in PAUP*, version 4.0b10 [40]. The null hypothesis of the SH test is that none of the trees evaluated has a likelihood that differs significantly from any other. Rejecting the null hypothesis indicates a significant difference in likelihood scores, and thus in tree topologies [49].

For a pair of trees defined a priori, the SH test computes the difference in their likelihoods (Δ). This difference is compared with the null distribution of likelihood scores, obtained by building trees from character data generated by iterative bootstrap resampling with replacement of the nucleotide sites. A computationally efficient optimization (RELL) may be applied, which simply adds together per-site likelihoods over the resampled sites. Otherwise, the tree parameters are optimized on the resampled data (FULL). The resampled likelihood differences are denoted Δ′i
 MathType@MTEF@5@5@+=feaafiart1ev1aaatCvAUfKttLearuWrP9MDH5MBPbIqV92AaeXatLxBI9gBaebbnrfifHhDYfgasaacH8akY=wiFfYdH8Gipec8Eeeu0xXdbba9frFj0=OqFfea0dXdd9vqai=hGuQ8kuc9pgc9s8qqaq=dirpe0xb9q8qiLsFr0=vr0=vr0dc8meaabaqaciaacaGaaeqabaqabeGadaaakeaacuqHuoargaqbamaaBaaaleaacqWGPbqAaeqaaaaa@2FA5@, where *i *indexes the replicate, and they are subsequently transformed by subtracting the mean resampled difference <Δ'>, a procedure called centering. The original difference in likelihoods is compared with the null distribution in a one-tailed, non-parametric manner, whereby the rank of Δ is evaluated against the centered, sorted Δ' distribution. If the rank of Δ is found to lie outside the interval of the null distribution between 0 and the (1-α) × 100 percentile, the difference in likelihoods is significant with (1-α) × 100% confidence, and the null hypothesis is rejected in favor of the alternative. (The acceptable type I, or false positive, error rate per test is denoted α.)

Here the tree topologies are ML phylogenies that represent different regions of the HCV genome. The reference alignment of 38 HCV whole-genome sequences representing 18 confirmed subtypes (Table 1) was obtained from the LANL HCV database [50]. We conducted SH tests with data from the 5' UTR, the Okamoto region of NS5B, and whole genome. Topologies were paired such that the ML tree Tx∗
 MathType@MTEF@5@5@+=feaafiart1ev1aaatCvAUfKttLearuWrP9MDH5MBPbIqV92AaeXatLxBI9gBaebbnrfifHhDYfgasaacH8akY=wiFfYdH8Gipec8Eeeu0xXdbba9frFj0=OqFfea0dXdd9vqai=hGuQ8kuc9pgc9s8qqaq=dirpe0xb9q8qiLsFr0=vr0=vr0dc8meaabaqaciaacaGaaeqabaqabeGadaaakeaacqWGubavdaqhaaWcbaGaemiEaGhabaGaey4fIOcaaaaa@3072@ inferred from the data of region *x *(either the 5' UTR or Okamoto region) was compared with the ML tree Ty∗
 MathType@MTEF@5@5@+=feaafiart1ev1aaatCvAUfKttLearuWrP9MDH5MBPbIqV92AaeXatLxBI9gBaebbnrfifHhDYfgasaacH8akY=wiFfYdH8Gipec8Eeeu0xXdbba9frFj0=OqFfea0dXdd9vqai=hGuQ8kuc9pgc9s8qqaq=dirpe0xb9q8qiLsFr0=vr0=vr0dc8meaabaqaciaacaGaaeqabaqabeGadaaakeaacqWGubavdaqhaaWcbaGaemyEaKhabaGaey4fIOcaaaaa@3074@ from data of region *y *representing each other region (either 5' UTR, Okamoto region, polypeptide, or whole genome, provided *y *≠ *x*), yielding the likelihood difference Δ ≡ *L*_*x*_(Tx∗
 MathType@MTEF@5@5@+=feaafiart1ev1aaatCvAUfKttLearuWrP9MDH5MBPbIqV92AaeXatLxBI9gBaebbnrfifHhDYfgasaacH8akY=wiFfYdH8Gipec8Eeeu0xXdbba9frFj0=OqFfea0dXdd9vqai=hGuQ8kuc9pgc9s8qqaq=dirpe0xb9q8qiLsFr0=vr0=vr0dc8meaabaqaciaacaGaaeqabaqabeGadaaakeaacqWGubavdaqhaaWcbaGaemiEaGhabaGaey4fIOcaaaaa@3072@) - *L*_*x*_(Ty∗
 MathType@MTEF@5@5@+=feaafiart1ev1aaatCvAUfKttLearuWrP9MDH5MBPbIqV92AaeXatLxBI9gBaebbnrfifHhDYfgasaacH8akY=wiFfYdH8Gipec8Eeeu0xXdbba9frFj0=OqFfea0dXdd9vqai=hGuQ8kuc9pgc9s8qqaq=dirpe0xb9q8qiLsFr0=vr0=vr0dc8meaabaqaciaacaGaaeqabaqabeGadaaakeaacqWGubavdaqhaaWcbaGaemyEaKhabaGaey4fIOcaaaaa@3074@), where *L*_*x*_(Ty∗
 MathType@MTEF@5@5@+=feaafiart1ev1aaatCvAUfKttLearuWrP9MDH5MBPbIqV92AaeXatLxBI9gBaebbnrfifHhDYfgasaacH8akY=wiFfYdH8Gipec8Eeeu0xXdbba9frFj0=OqFfea0dXdd9vqai=hGuQ8kuc9pgc9s8qqaq=dirpe0xb9q8qiLsFr0=vr0=vr0dc8meaabaqaciaacaGaaeqabaqabeGadaaakeaacqWGubavdaqhaaWcbaGaemyEaKhabaGaey4fIOcaaaaa@3074@) is the likelihood of the ML tree from region *y *evaluated with data from region *x*. We randomly resampled 10,000 replicate data sets for each pair of trees and compared the original difference in likelihoods with the null distribution that resulted. The type I error rate was reduced to accommodate six hypothesis tests (α = 0.05/6 = 0.00833). This reduction preserves the experiment-wide false-positive rate by making each comparison more stringent.

### Consistency and homoplasy indices

To understand better phylogenetic inconsistencies over the HCV genome, we computed the character consistency index (CI) for each site in PAUP with the whole-genome phylogeny, and summarized CI with a moving-window (running) average over 100, 300, and 500 nt. The 100 nt window size was used subsequently because it allows for clear visualization of the 342 nucleotides that constitute the 5' UTR. Because the consistency and homoplasy indices (HI) are complementary (CI+HI = 1), character consistency is high when homoplasy is low, and vice versa. Thus, we expect lower homoplasy to result from fewer informative sites. Further, homoplasy decreases rapidly with decreasing substitution rates. To control for variation in the number of informative sites across the genome, we rescaled the homoplasy index against the square of the proportion of informative sites in the window region. This was done because, in the limit of short branch lengths, the number of informative sites should be proportional to the substitution rate *r*, while the number of homoplasies should be proportional to *r*^2^. The result was subsequently normalized against the maximum, to facilitate comparison with the proportion of informative sites. As a result, if all parts of the HCV genome are equally informative, one can expect the rescaled homoplasy index to be roughly constant over the viral genome.

## Competing interests

The author(s) declare that they have no competing interests.

## Authors' contributions

All authors contributed equally to the conceptualization, experimental design, data analyses, and narrative presented herein.
